# Analysis of Scientific Collaboration Networks among Authors, Institutions, and Countries Studying Adolescent Myopia Prevention and Control: A Review Article

**Published:** 2019-04

**Authors:** Wenwen WU, Yaofei XIE, Xiangxiang LIU, Yaohua GU, Yuting ZHANG, Xinlong TU, Xiaodong TAN

**Affiliations:** 1. School of Health Sciences, Wuhan University, Wuhan 430071, Hubei Province, China; 2. School of Public Health and Management, Hubei University of Medicine, Shiyan 442000, Hubei Province, China

**Keywords:** Social network analysis, Collaboration, Myopia, Prevention and control

## Abstract

**Background:**

Studies related to the prevention and control of myopia in adolescents have increased rapidly, but only a few have measured the levels of scientific collaboration among authors, institutions and countries in this field. Thus, in this study, we aimed to reveal the status and levels of scientific collaboration in this field.

**Methods:**

The research population included all published papers in the field of adolescent myopia prevention and control indexed in the Web of Science databases from 1997–2016. The co-authorship networks were drawn using SATI (Statistical Analysis Toolkit for Informetrics), Ucinet and VOS viewer (Visualisation of Similarities viewer). Active authors and some measures of co-author network, including degree centrality, closeness, betweenness, density and diameter, were also assessed.

**Results:**

Overall, 610 records were obtained, and a number of publications developed through an increase in different collaboration types, with cooperation among authors and institutions as the most apparent ones. The top ten active authors and institutions were identified. The density of cooperative networks of the top 70 authors and the first 69 institutions were 0.043 and 0.011, respectively, with corresponding diameters of five and six, respectively. Seven distinct clusters formed the cooperation network among 38 countries. The top three clusters were centered in China, the United States and Australia, also identified as the most productive countries.

**Conclusion:**

The flow of information is slow and the collaboration among authors and institutions in the network are not close enough. Thus, multiple collaboration types should be encouraged in this field, especially among countries.

## Introduction

Myopia is the main cause of preventive blindness worldwide, especially in adolescents (1, 2). Thus, it is one of the main priorities among the five projects under the ‘Vision 2020 Action’ launched by WHO (3). In recent years, the incidence of myopia has increased rapidly worldwide (4), especially among adolescents in East and Southeast Asia (5, 6). The prevalence of myopia among adolescents is at 96.5% (7) in South Korea, 81.6% (8) in Singapore and 95.5% (9) in Shanghai.

Myopia not only affects adolescents’ school performance and future career choice (10) but also causes glaucoma, cataract and other serious complications (11). Thus, many researchers have devoted themselves to gaining a more in-depth understanding of the prevention and control of adolescent myopia (12, 13).

Collaborative research networks can help other researchers expand their field of research or join groups conducting related studies. Bibliometric studies of scientific collaboration have been conducted in various fields (14, 15), providing different levels of cooperation frequency in research practice. One of the methods used to study such collaboration is the co-authorship network analysis, which focuses on finding patterns of contacts or interactions between social actors. Author, country, and institution are the subjects of cooccurrence relationship; thus, analyzing their cooccurrence relationship can better reflect the truth of scientific research and academic communication, because the cooperation of authors, institutions and countries can measure the cooperation at different levels (15).

However, to date, no bibliometric analysis of scientific literature in myopia prevention and control had been carried out and published. As such, this study aimed to describe the diversity of cooperation among authors, institutions, and countries in the study of adolescent myopia prevention and control. Specifically, for adolescent myopia prevention and control research, our main goal is to explore the following content: firstly, analysing the overall status of collaborative research among authors, institutions and countries; secondly, determining the institutions and authors at the core of the cooperative research network; and thirdly, identifying countries that have a strong cooperative relationship.

## Materials and Methods

The search for papers to be included in the analysis was conducted in one day (Sep 25, 2017) to avoid bias resulting from daily updating in the database. The Web of Science Core Collection annually collects a large number of journals and records each publication, including bibliographic information (i.e., author, institution and country or region), which we used to locate publications. All papers published within the period of 1997–2016 were evaluated. Search terms included combinations of terms, such as ‘adolescent’, ‘children’, ‘student’, ‘myopia’, ‘myopic’, ‘prevention’, ‘control’ and ‘management’. Literature types, such as meeting abstracts, letters, correction, news item, book chapter, retracted publication, editorial material, non-English literature and repeated articles, were excluded. To ensure reliability, profile information of each included article was extracted by two independent reviewers, resulting in a reliability check of 100% of the selected abstracts. A search query that was used for data extraction from Web of Science database looked like this: TS= ((adolescent myopia OR children myopia OR student myopia OR adolescent myopic OR children myopic OR student myopic) AND (prevention OR control OR management)).

Social network analysis (SNA) is a method of structural analysis applied in many research fields. It focuses on relationship research and is mainly used to describe and measure relationships and information between individuals (16). SNA has been proven to be effective in studies on scientific collaboration network (17, 18). The same method is used in the current study.

To analyze and identify critical issues, we used SATI (Statistical Analysis Toolkit for Informetrics) (ver. 3.2) to build the co-occurrence matrix (19) and transformed the data format with Ucinet 6.0 (20) to finally obtain co-occurrence mapping. VOS viewer (Visualisation of Similarities viewer) software (ver. 1.6.6) was employed to draw the co-country (region) maps by using literature title packets (21). Excel 2016 (Microsoft, Redmond, DC, USA) and Netdraw (ver. 2.118) were also used in the research. In addition, some measures of our network, including degree centrality, betweenness centrality, closeness centrality, density, and diameter, were evaluated (22). Degree centrality refers to the number of neighbors to a node in the network (15). In this case, the greater its connection to other nodes in the network, the more important is the node.

Betweenness centrality refers to the number of the shortest paths passing through a given node (23). The higher the betweenness centrality of the node, the greater the ability to control the information passed between the other nodes. The closeness centrality is used to measure the distance of one node to other nodes in a network. Nodes with high closeness centrality obtain information better than other nodes or tend to have a more direct influence on other nodes (16). Density is calculated through the actually observed ties divided by all possible ties whose value is between 0 and 1 (24). Density values tend to reach 0 in sparse networks, and close to one in tightly connected networks (24). The diameter represents the longest measuring distance in a connected network; it shows the number of steps required from one side of the network to the other (16).

### Ethical considerations

This study did not require any ethical consideration as it does not include any human or animal to be the object of study.

## Results

A systematic search for publications on adolescent myopia prevention and control retrieved 624 articles in Web of Science Core Collection, excluding one duplicate. After further screening of titles and abstracts, 9 editorial materials, 4 letters and a meeting abstract were removed, leaving 610 eligible papers.

### The scale and overall trend of collaborative research

**Fig. 1:**
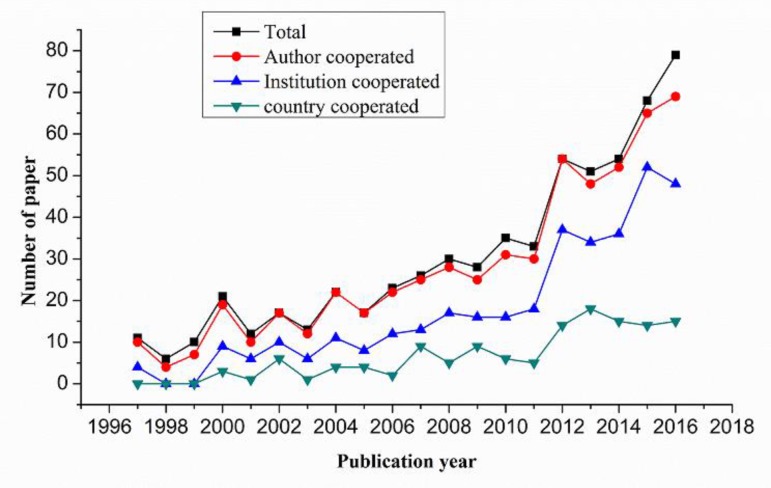
Numbers of papers on adolescent myopia prevention and control by collaboration type between 1997 and 2016

Figure 1 shows the number of publications issued annually and the number of papers published through collaboration with authors, institutional cooperation and country (region) cooperation. The number of papers, co-authors, co-institutions and country (region) cooperative papers has increased significantly from 1997 to 2016, particularly after 2011. In general, the total number of published articles since 1997 has increased more than six-fold, from 11 in 1997 to 79 in 2016; the institutional cooperation increased more than five-fold, the author cooperation increased by twelve-fold and the country (region) cooperation increased by fifteen-fold.

**Fig. 2:**
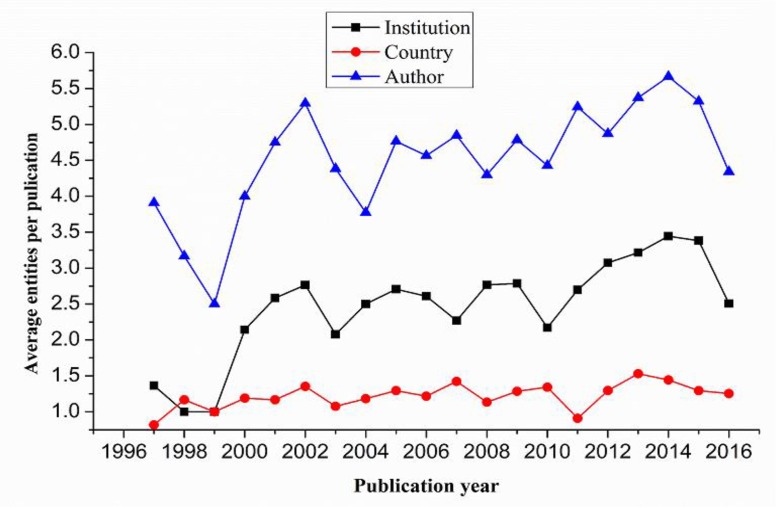
Average number of different entities per paper

Fig. 2 reveals the average number of authors, institutions and countries per article from 1997 to 2016. The average number shows a gradually increasing trend.

The increase in number of authors was from 3.91 to 4.34, from 1.36 to 2.51 for institutions and from 0.82 to 1.25 for countries per paper. Overall, the rates of cooperation among authors, institutions and countries were 93%, 57.9% and 21.5%, respectively.

**Fig. 3:**
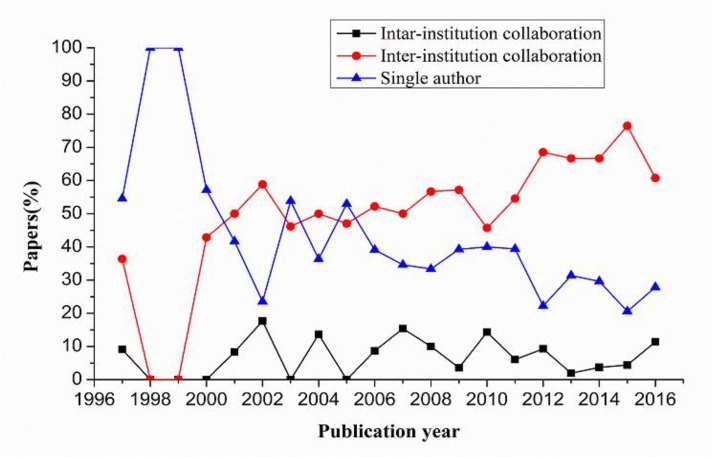
Percentage of different collaboration types

In general, the number of SCI journal papers produced by institutional cooperation is the largest (accounting for 56.6%), followed by papers generated through intra-institutional collaboration (accounting for 36.1%) and papers produced without collaboration (accounting for only 7.4%). Figure 3 shows the percentage of papers studied in each of the different institution collaboration types and their changes over time. The percentages of single-author papers have decreased by 26.7% from 1997 to 2016, whereas that of institution-collaborated papers increased by 24.4%. The percentage of papers produced through single authorship has always been higher than that of institutional collaboration from 1997 to 2000 but decreased after 2006.

### Authors’ collaborative research

Results of scientific research are published in the form of papers, and the status of co-authorship in papers reflects the collaboration among authors. Researchers who study the growth of co-authorship articles produced by multiple authors regard co-authorship of papers as a significant scientometric indicator of researching on cooperation among authors (25).

**Fig. 4:**
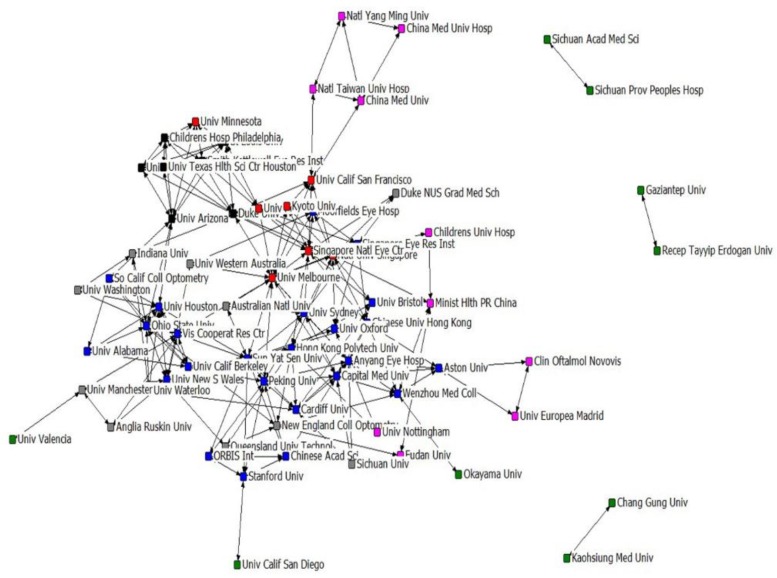
The structure map of the author collaboration network

More important researchers were expected to have published more articles, thus scholars who published more than four articles were included in the co-authorship networks. Overall, 75 researchers with 371 co-authored experiments meet this condition. Five authors not cooperated with other authors were excluded. The research collaboration network between authors is shown in Fig. 4.

Each node of the figure represents an author, and the connections among the nodes represent the collaboration relationships among authors. The weight of a link indicates the number of publications co-authored by two scholars. In this author’s collaboration network, the highest degree centrality of Allen, Peter M. and O'Leary, Daniel J. was 5.83, indicating that they had 5.83 collaborators and that they played a pivotal role in the co-authorship network. Saw, Seang Mei obtained the highest betweenness centrality manifesting that he had the ability to control collaborative relationship and that he possessed and controlled a large number of research resources. In collaborative network, the closer the distance between one author and the other, the easier it is to exchange information and build cooperative research relationship. Saw, Seang Mei and Mitchell, Paul had the highest closeness centrality, which manifested that they had the most opportunity to exchange information and establish a cooperative relationship with other authors (Table 1). Furthermore, we assessed the density and diameter of co-authorship network, which were 0.043 and 5, respectively.

**Table 1:** Top 10 authors on centrality measures in collaborative network

***Degree***	***Score***	***Betweenness***	***Score***	***Closeness***	***Score***
Allen, Peter M	5.83	Saw, Seang Mei	72.00	Saw, Seang Mei	1.85
O'Leary, Daniel J	5.83	Mitchell, Paul	56.00	Mitchell, Paul	1.85
Guo, Xiangming	5.56	Gao, Yang	48.00	Liu, Luo-Ru	1.84
Wang, Panfeng	5.56	Congdon, Nathan	48.00	Li, Shi-Ming	1.84
Xiao, Xueshan	5.56	Manny, Ruth E	42.00	Li, He	1.84
Li, Shiqiang	5.56	Davitt, Bradley V	40.00	Li, Si-Yuan	1.84
Jia, Xiaoyun	5.56	Qu, Jia	26.00	Kang, Meng-Tian	1.84
Zhang, Qingjiong	5.56	Tan, Donald	14.00	Qu, Jia	1.84
Price, Holly	5.39	He, Mingguang	13.00	Tan, Donald	1.84
Rae, Sheila	5.39	Liu, Luo-Ru	3.60	Pan, Chen-Wei	1.84

### Institutions’ collaborative research

**Fig. 5:**
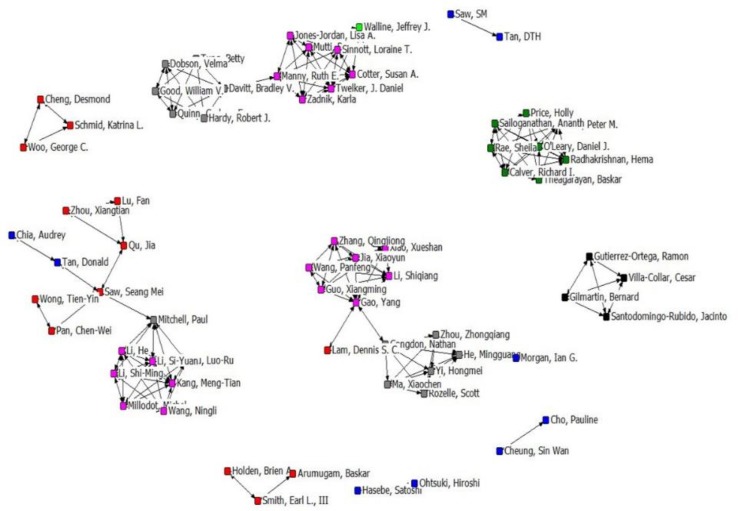
The structure map of the institutional collaboration network

It would be helpful for us to study the academic information exchange mode in scientific collaboration by analyzing the institutional relationship network of research collaboration (26). Among 610 papers, 353 documents were produced by inter-institution cooperation and 45 papers produced by intra-institutional cooperation. These papers covered 480 actual institutions and the total appearing frequency of institutions is 1674. The largest collaboration in the sample of this study involved 22 institutions. We deleted five institutions not cooperated with other institutions and eventually selected the top 69 institutions with appearance frequencies excessing five to form a map visualizing the structure of institution’s collaboration network in the field of adolescent myopia prevention and control during 1997 to 2016 (Fig. 5). The size of the node indicated centrality in collaboration network. The network’s density is 0.011 and diameter is 6. Table 2 lists the top ten institutions in the adolescent myopia prevention and control research dataset based on three measures of centrality: degree, betweenness, and closeness centrality. The Smith-Kettlewell Eye Research Institute had the highest degree centrality and that the University of Melbourne had the highest betweenness centrality and the lowest closeness centrality. Furthermore, the Smith-Kettlewell Eye Research Institute is found to be the most critical institution in the cooperation network and enjoys a high level of cooperation with the University of Melbourne.

**Table 2:** Top 10 institutions on centrality measures in collaborative network

***Degree***	***Score***	***Betweenness***	***Score***	***Closeness***	***Score***
The Smith-Kettlewell Eye Research Institute	3.19	University of Melbourne	397.14	the University of Melbourne	12.67
Saint Louis University	3.19	Sun Yat-sen University	262.38	National University of Singapore	12.50
University of Pennsylvania Health System	3.08	National University of Singapore	255.13	Sun Yat-sen University	12.48
University of Texas Health Science Center at Houston	2.59	Duke University	238.41	Singapore National Eye Centre	12.41
University of Arizona	2.35	University of California, San Francisco	228.00	Duke University	12.27
Children's Hospital of Philadelphia	2.09	University of New South Wales	150.88	The Hong Kong Polytechnic University	12.23
Orbis International	1.82	The Hong Kong Polytechnic University	140.97	The Chinese University of Hong Kong	12.21
Stanford University	1.71	The University of Arizona	119.22	Peking University	12.18
Chinese Academy of Sciences	1.58	Aston University	118.00	Capital Medical University	12.18
European University of Madrid	1.56	The Chinese University of Hong Kong	117.56	Anyang Eye Hospital	12.16

### Countries’ (regions’) collaborative research

**Fig. 6:**
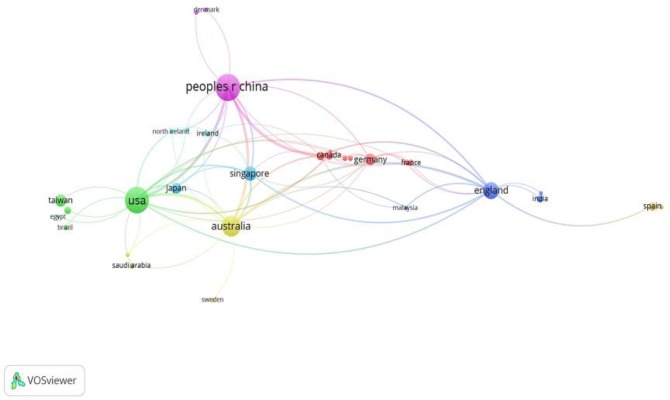
Collaboration network visualization of countries (regions)

**Fig. 7:**
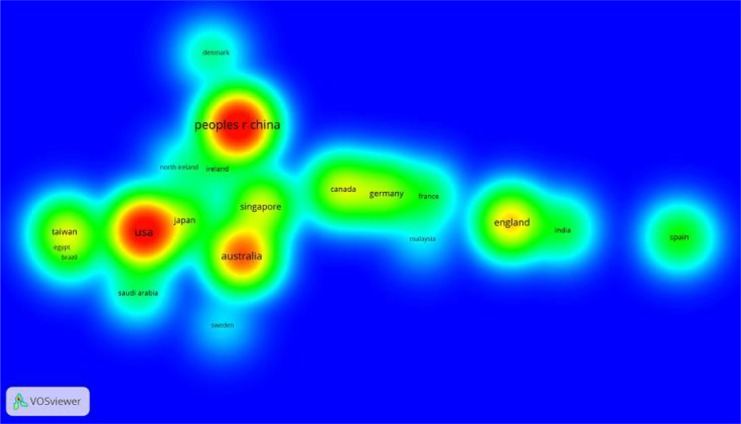
Density view of co-authorship network of countries (regions)

The research articles produced by international cooperation have greater influence (26). Our dataset involved 53 countries, excluding 15 countries whose paper production had been less than two in the past 20 years. Using the rest of the 38 countries, we constructed a collaboration network through the VOS viewer, which helped depict the relationships between these countries. Figures 6 and 7 are visual presentations of the collaborative networks among different countries (regions). In Fig. 6, the size of the node represents the number of papers produced in that country or region, wherein the thickness of the links is positively correlated with the strength of the collaboration (27). The colors represent the collaboration clusters and each color represents a separate cluster (27). Hot spots are colored in red and appear lighter as they go farther away from the center of gravity. The thickness of the links represents the strength of collaborations between the countries (regions) it connects. In Fig. 7, seven major clusters can be distinguished: the largest one gathering around China, the next one around the USA and the other clusters gathering around Australia, England, Singapore, Germany and Ireland. The highest density in the network belonged to China, USA and Australia.

## Discussion

The increase of myopia incidence is related to many factors, such as genetic (28), environmental risk factors (1) and unhealthy lifestyles (29). The early onset of myopia is reported to cause faster development and higher possibility of occurrence in adulthood (30). Therefore, prevention and control of myopia in adolescence are of great significance. We can try to do more intervention research on the risk factors that are controllable or changeable. Research collaboration is an important way to improve the overall strength of research and enables researchers to supplement each other's strengths and share information (31).

We found that, in the past 20 years, the total number of papers in the field of prevention and control of myopia in adolescents, as well as the total number of co-authored articles, increased especially since 2011. Previous studies in other research fields showed a similar trend (15, 32).

The average number of authors, institutions and countries (regions) per paper has also increased over time. In general, 93.0% of the publications are co-authored by more than one author, whereas 57.9% of the publications have been co-authored by a number of institutions over the past 20 years. On the one hand, researchers not only benefit from knowledge exchange but also save on research costs due to the sharing of information, technology and resources (33). On the other hand, as research goes deeper, it becomes increasingly difficult to achieve a great breakthrough through a single person or institution, which forces researchers in adolescent myopia prevention and control field to cooperate with others.

The above analysis indirectly indicates that scientists and research institutions attach importance to cooperation; thus, researchers will have great propensity to cooperate in the adolescent myopia prevention and control field.

As social networks are developed by adding new nodes and links, and the new knots are connected to old high central knots based on the principle of preferential connection (34), scholars or institutions with high degree centrality can play a vital role in the development of co-authorship networks. From the results of degree centrality analysis, Allen, Peter M. (5.83) and O'Leary, Daniel J. (5.83) emerged as the top researchers with the most frequent collaborative activities, followed by Guo, Xiangming, Xiao, Xueshan, Li, Shiqiang, Jia, Xiaoyun and Zhang, Qingjiong. The Smith-Kettlewell Eye Research Institute and Saint Louis University are institutions with the most frequent cooperative activities. Hence, Allen, Peter M. and O'Leary, Daniel J. had the highest number of opportunities to communicate with other members of the network and had the greatest ability to build collaborative teams in the growth and dynamics of network, as well as the Smith-Kettlewell Eye Research Institute and Saint Louis University.

Our betweenness results showed that Saw, Seang Mei and the University of Melbourne had the highest scores, indicating that they play a good mediating role in the cooperative network and control in the flow of information.

The closeness centrality measures the distance of one member from the other members in a network (16). In this study, two authors (Saw, Seang Mei and Mitchell, Paul) and one institution (University of Melbourne) had the highest closeness centrality, indicating that they receive information faster than others because of fewer intermediaries among them.

Whether author collaboration or institutional collaboration network, network density is very low, which means that the cooperation among the authors and the relationship between the institutions are not tight enough. The low network diameter also implies that the communication between the authors and the information exchange between the institutions is slow.

Visualization analysis of cooperation among countries suggests that China, the USA and Australia are the most productive countries and are also the hotspots for the study of adolescent myopia prevention and control. Compared with other studies indicating that collaborative countries are often geographically interrelated (32), our results present conflicting outcomes considering the distribution of seven major clusters. In the co-authorship network of countries, the developed countries occupy the vast majority.

Although our study is one of the first attempts to systematically describe the research collaboration in this field, it also has some limitations. Firstly, in order to guarantee the homogeneity of the research samples, the books, meeting records, reports, letters, editing materials and non-English articles were excluded in the analysis, which may bring about the issue of incomplete information. Secondly, our search terms may not be used in the title of some relevant research and cannot be retrieved by our method. Thirdly, since no database is perfect and some might have bias by over-representing journals using the English language, bibliometric results should always be considered with caution (35).

## Conclusion

This study provides a systematic description of collaboration at the levels of author, institution and country (region) in the research on adolescent myopia prevention and control. Although the number of publications has been increasing, the information flow is slow and no close collaboration occurs among the authors as well as among institutions in the network. Multiple collaboration types should thus be encouraged in this field, especially among countries. Moreover, middle and low-income countries need to strengthen cooperation with developed countries.

## Ethical considerations

Ethical issues (Including plagiarism, informed consent, misconduct, data fabrication and/or falsification, double publication and/or submission, redundancy, etc.) have been completely observed by the authors.
